# The costs of avoidable injury from childhood cancer: Litigate or mediate?

**DOI:** 10.1177/00258172221099077

**Published:** 2022-09-22

**Authors:** David A Walker, Jonathan Punt

**Affiliations:** 1 Emeritus Professor in Paediatric Oncology, Children’s Brain Tumour Research Centre, University of Nottingham, NG7 2UH; 2 Retired Barrister (Formerly of No 5 Barristers Chambers, Birmingham-London-Bristol-Leicester, UK)

**Keywords:** Child cancer claims, child brain tumour claims, alternative conflict resolution, public awareness campaigns, health economic consequences

## Abstract

We write as experienced paediatric practitioners who have been involved in medico-legal proceedings where cases related to childhood cancer practice have featured frequently. We will use the service evaluation of Professor David A Walker’s last 35 cases, where all but seven concerned children with tumours of the brain or spine to illustrate the concerns that families raise. We refer to the evidence from the HeadSmart programme (www.headsmart.org.uk), which seeks to accelerate diagnosis by raising awareness of the disease and symptoms. We use the experience of Dr Jonathan AG Punt to illustrate the legal issues that apply and explain the way that significant quantum calculations are applied to cases of this type. The current move by NHS Resolution to explore the expanded role of mediation will be discussed and the need for research to explore the precise way that mediation could be developed to offer an alternative approach to conflict resolution.

## Introduction

Mediation is proposed as an alternative mechanism for conflict resolution; evidence to compare its effectiveness, acceptability and impact is lacking. This area of practice may offer a suitable field for such studies.

We write as an experienced paediatric oncologist (DAW) and a dually qualified paediatric neurosurgeon and barrister (JP) who have been involved in medico-legal proceedings where cases related to childhood cancer practice have featured frequently. We will use the service evaluation of DAW’s last 35 cases over the past five years, where all but seven concerned children with tumours of the brain or spine, to illustrate the concerns that families raise. We will use the experience of JP to illustrate the legal issues that apply and explain the way that significant quantum calculations are applied to cases of this type. The current move by NHS Resolution to explore the expanded role of mediation will be discussed.

## Practice review

(See Table 1 Supplementary Material.) Of the 35 consecutive case reports over the past five years that are the focus for this review, 27 were due to concerns about diagnostic delay, 7 were concerning specialist clinical management including treatment complications. Paediatricians were the largest target group of practitioners, followed by the referral network(s), radiologists, paediatric surgeons and other sub-specialists seeing children. The commonest proposed breaches were related to initiating investigations, particularly scans and the accuracy of their reporting. Causation was proposed for sudden death, shortened life expectancy, focal and generalised brain damage and spinal injury resulting in paraplegia and incontinence. Four cases presenting with sudden, unexpected deaths were due to mediastinal lymphoma, overwhelming sepsis complicating undiagnosed Hodgkin’s disease, acute or chronic hydrocephalus due to cerebellar brain tumour and acute presentation of hypothalamic astrocytoma. This number of cases is a notable case load for a single expert, given that there are only 2000 cases of childhood cancer and 450 new cases of childhood brain tumour diagnosed each year in the UK. Cure rates for childhood cancers, including brain tumours, in the UK are about 70%, indicating that disabled survivors can expect a long life.

## Clinical expert role

As an independent expert, contributing to the processes in report writing is the first step; involvement in expert meetings with solicitor and counsel is common and generally most helpful to identify the key medical issues. It is notable that none of these cases were taken to court for a judicial determination on matters of liability. The expert medical opinions as to possible breaches of duty and any causative effect are therefore advanced, but untested at trial and therefore unproven in law. Any settlements of damages that had been reached between the parties would have required the approval of the court on account of the claimant being a child or older “*protected party*” (Civil Procedure Rules 1998, Part 21, rules 21.1(2) and 21.10) and thereby lacking capacity under the Mental Capacity Act 2005. Feedback with regard to any civil litigation for DAW practice review was not disclosed in all cases by the instructing solicitors.

## Factors contributing to breach of duty in diagnosis of brain and spinal tumours

The three commonest anatomical locations for diagnostic difficulties in brain and spinal tumour are mirrored in the proportions in the case review. Specifically, tumours presenting in the posterior fossa (40%), involving the cerebellum and brainstem, and the hypothalamic region involving the optic pathways (20%), and the spinal column involving spinal and paraspinal tumours (12%). The experience of these cases highlights the factors that contributed to the proposed breaches of duty.

**Cases involving cerebellar and hypothalamic tumours** featured evolving symptoms of raised intra-cranial pressure (RICP), notably headaches and vomiting, due to 4th ventricular obstructive hydrocephalus or hypothalamic mass. Practitioners were distracted by their fluctuating nature, favouring gastric explanations for vomiting despite negative tests, and proposing psychological or psychiatric explanations. Failed weight gain or weight loss associated with anorexia was attributed to somatic or psychiatric causes without brain scanning or endocrine studies, when they occurred in hypothalamic tumours. Sustained trends in the whole history were often overshadowed by the problem on the day, assessed by a variety of practitioners. In infants and children under two years, arrest of developmental progress was not identified as a reason to investigate the brain. Measurements of head circumference in infants and young children were incorrectly interpreted or not plotted on centile charts. Neurological examinations were superficial and did not always include the optic fundi to identify papilloedema and/or optic atrophy. Assessments of growth with measurement of height, weight and assessment of pubertal status in primary care were omitted. Visual signs were not noted or investigated. Practitioners misinterpreted hydrocephalic attacks for epilepsy. Overall, practitioners frequently failed to reconsider diagnostic options and developed closed thinking. Parental requests for brain scans were discounted by the medical practitioners. Where scans were ordered, some were not followed up; computed tomography (CT) scans without contrast were vulnerable to misinterpretation, as were magnetic resonance (MR) brain and spinal scans, when reported by general radiologists. Delays led to accumulated brain injury. Where death occurred, appropriate brain imaging would probably have saved lives.

**Cases involving spinal cord compression** occurred from infancy to early adulthood due to a range of intra-spinal and paraspinal tumours. In infancy, reduced movement of lower limbs was not identified as a reason to investigate. Persistent and significant back pain, particularly at night, was not recognised as a symptom to trigger imaging in a child. Where sacral or paraspinal masses were found on ultrasound, intraspinal extension was not suspected and MR scans to examine spinal cord integrity were not ordered. Examination of lower limbs with record of reflexes were incompletely conducted or recorded and not reviewed during periods of observation. Specialist referrals accumulated delays in multi-disciplinary team (MDT) meeting schedules, planning for biopsy and initiation of treatment, whilst symptoms persisted and function deteriorated. Consultant to consultant communication during referral did not occur. Rare benign vascular tumours fluctuated in childhood and progressed later in adult life causing neural injury. There were no hospital or national protocols for management of suspected spinal cord compression in children.

### National awareness campaigns

Over the past decade there have been three public and professional awareness programmes concerned with the symptomatology of childhood cancers^5-8^: (a) HeadSmart for brain tumours (www.headsmart.org.uk; Figure 1a), (b) ChildCancerSmart (Figure 1b) for childhood cancer risk and c) the Grace Kelly Childhood Cancer Trust illustrating the symptomatology of childhood malignancies (Grace Kelly; Figure 1c). These web-based tools offer child-centred risk categories and symptom guides which, together, support the view that the risk of cancer for the child and young person (1 in 320 by age 20; Figure 1d) exceeds the risk of a health professional seeing a case in their practice. Together these may challenge the Bolam defence where the extreme rarity of a condition may be argued to relieve a practitioner of the duty to recognise it as a diagnostic possibility. In paediatric practice, the child’s needs are considered paramount;^9^ the practitioner seeing children has a duty to be aware of the health risks for children.

**Figure 1a.**
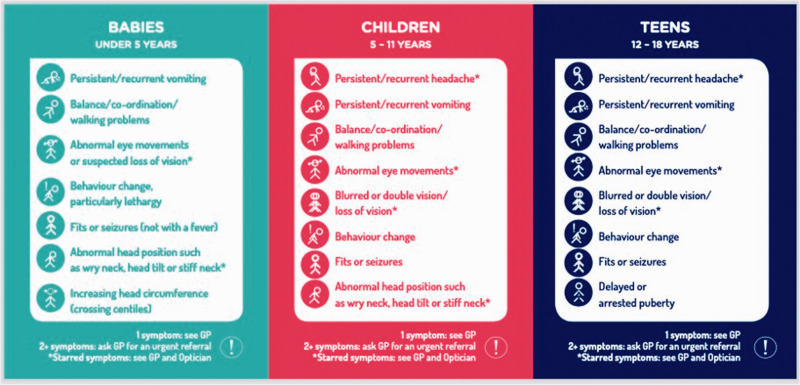
HeadSmart Brain Tumour Age Related Symptom Checklist distributed as part of national campaign in UK 2011 to present day.^9^

**Figure 1b.**
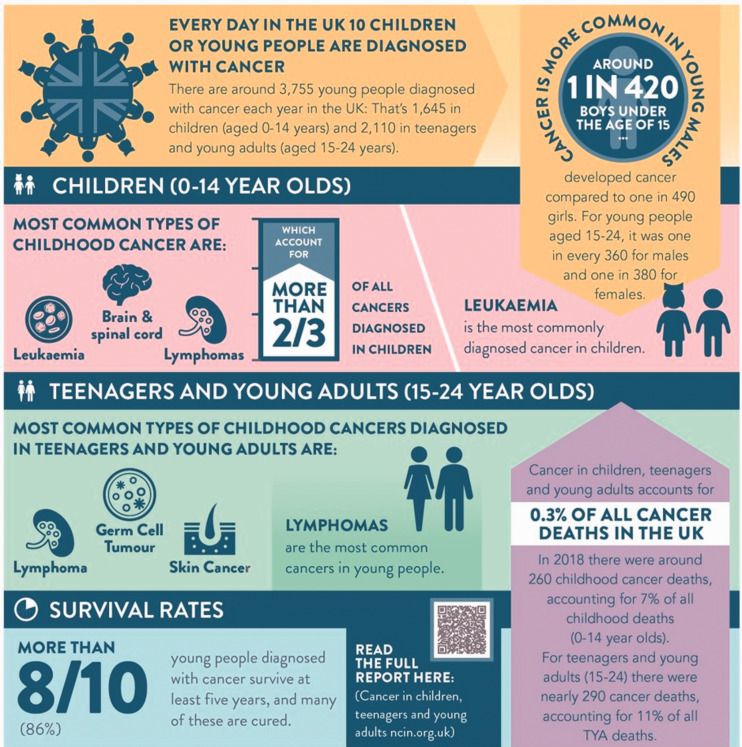
ChildCancerSmart infographic describing population risks for childhood cancer.^4^

**Figure 1c.**
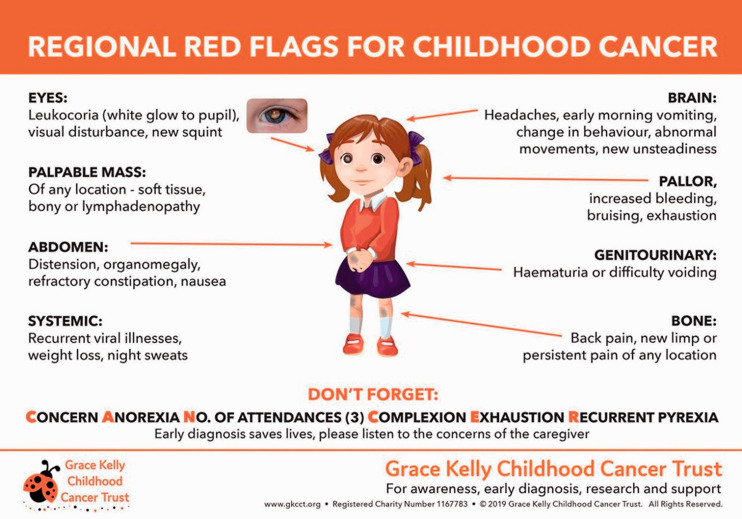
The Grace Kelly Childhood Cancer Trust infographic describing symptomatology of childhood cancers (www.gkcct.org).

**Figure 1d.**
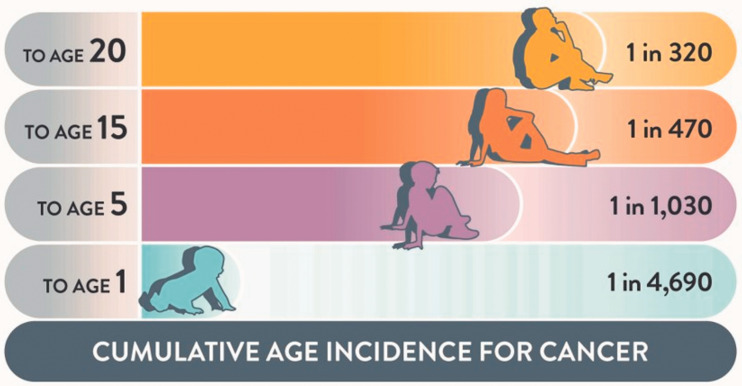
The age related risks of cancer across childhood and early adulthood.^9^

## Multi-disciplinary team judgment

Not all cases were focused on delay in diagnosis. Complex patient management involving MDTs are specified as part of all NHS cancer practice standards. Non-compliance with MDT meeting requirements for core membership during case discussion undermines the collective protection of a group decision. If a core team member is not present the expert team lacks that expertise and their decisions may be challenged. Record keeping of these meetings is critical. Furthermore, the MDT decision to operate must be associated with compliance with informed consent by the surgeon for the surgery specified by the MDT. Current MDT decisions are being informed by new complex molecular data overlaying the core pathological descriptions of tumour entities, upon which historical treatments and outcomes are based. Delays resulting from prolonged waits for molecular results and rejecting histological assessments with rare molecular observation, because a targeted drug might be available, is an area of new practice which can be judged in retrospect to have been vulnerable to interpretation bias, especially where samples are deemed inadequate for formal pathological assessment. Such judgements were contributory to alleged breaches of duty of care by MDTs.

## Health economic consequences

The adverse effects of a child experiencing avoidable neurological sequelae in connection with a brain or spinal tumour go far beyond those that can be measured in pure health economic terms. The physical and psychological effects on the child will be lifelong, constraining prospects for the affected child fulfilling her/his innate potential in terms of education, work and personal relationships. The adverse effects will affect the child’s parents, and other family members, for the duration of their own lives. To reduce these consequences to a cold pecuniary evaluation may be regarded as adding insult to injury. However, as they are an essential and unavoidable component of civil litigation it is justifiable to include their assessment in considering the potential value of mediation. Indeed, the calculation of damages that may fall to be recovered in successful civil litigation, arising from alleged clinical negligence, are an approximate indicator of the economic consequences of such diseases. The loss to society of the contribution that the injured person would have made, but for the avoidable injury, is immeasurable, but is far from *de minimis.* A detailed description of how damages are classified and calculated is provided in the additional material. Examples of actual compensatory awards related to these types of injury are described and range from £2.6 million to £26 million. (See Supplementary Materials for detailed records of award in relevant cases.)

## Sudden death: alternative conflict resolution

Children can die from cancer presentations acutely. The cases included in the review are typical. Where a child does die as a consequence of delay, an inquest may be the mechanism for an inquiry into the circumstances contributing to the death. There are strict legal limitations as to the wording of a coroner’s determinations and conclusions. The Coroners and Justice Act 2009 prohibits them to be framed in such a way as to appear to determine any question of criminal liability on the part of a named person, or civil liability.^1^ A coroner may, however, conclude that the evidence adduced is sufficient to conclude that neglect contributed to the death. Neglect has a specific legal meaning in Coronial Law and does not equate to negligence in civil proceedings. Notwithstanding the legal constraints upon a coroner, the very fact that members of the deceased child’s treating team have been questioned in public and given evidence under Oath or Affirmation, and that the pertinent facts have been disclosed, may bring relief to families and provide the explanations they need. It can be time consuming and harrowing. The majority of coroners do not have medical qualifications and a family will require legal representation by an appropriately qualified solicitor or barrister if they, and the coroner, are to discover the full facts. Further, it is noted that a coroner may decide not to seek opinion evidence from independent expert witnesses. Where a coroner relies upon the treating clinicians in matters of opinion, as opposed to matters of factual evidence, there is a risk that the coroner will be misled. Further, some coroners will go to great lengths to restrict questions that they perceive as having the potential to lead to discovery of facts that could disclose negligence.^2^ On occasion, such coronial conduct has been at such a level to result in Judicial Review with determination in favour of the bereaved family.^2^ It is disappointing to note that such risk is real, rather than hypothetical, notwithstanding the statutory Duty of Candour required of doctors and nurses under the Health and Social Care Act 2008 (Regulated Activities) Regulations 2014,^3^ but regrettably not always adhered to in practice.^2^

Despite these provisos, an inquest does not always follow a death, as the coroner may choose not to investigate if the cause of death is recognised. Families can therefore be left feeling that there are unanswered questions and seek legal redress through civil litigation. It has been DAW’s practice, in cases where the child has died, to recommend requesting an internal Serious Untoward Incident Investigation (“SUII”) if there has been no coronial investigation. Indeed, it is not unusual for an SUII to have preceded an Inquest. It should be noted that a coroner is not bound in any way by the findings of an SUII and will reach her/his conclusion as to the extent to which the SUII, and/or its documentation, is adduced in evidence. In DAW’s experience as a clinical expert, the SUII normally identifies areas for improved practice. It offers the family a mechanism of redress using internal review processes, although the duty of candour to inform the family that an investigation is anticipated, or has concluded, is not always followed. From JP’s perspective this has not always been the experience with SUIIs, which by lacking thoroughness, candour, honesty, or any external independent opinion, have exacerbated the distress, anger and lack of faith on the part of the affected family.^2^

In such circumstances, mediation may prove to be an effective way of resolving conflicts, as the legal process of allocating liability and awarding compensation, where a child has died, are frequently misplaced and unaffordable. The clinical expert can play an important role by explaining and, if appropriate, supporting the interpretation of the clinical details in the SUII with the family concerned and suggest what can be expected of the Trust or practitioner to resolve the conflict. In other cases, where death has not occurred, mediation may also be adopted as a strategy for conflict resolution. We suggest that this might be appropriate:

- where there is dispute between parent/child and treating team as to management strategies, including issues of provision or withdrawal of therapies;
- where there has been an error, or perception of error, that may or may not be negligent in law, and there is dispute between the parent/child and treating team as to any connection with adverse consequences for the patient;
- where parent/child have indicated an intention of seeking legal advice as to perceived clinical negligence;
- where parent/child seek a non-pecuniary solution or remedy, either because no damages are likely to be obtainable, or out of personal choice;
- within the course of civil litigation, even as an alternative to the more usual Joint Settlement Meeting.

Direct contact in a safe and supportive environment between the family and the practitioners and their employing Trust can contribute to successful resolution. In addition, it offers a mechanism for settling damages, and can also provide non-pecuniary remedies, including frank acknowledgments of causative failures, apologies for same, and undertakings to take steps to reduce the risk of recurrence, all of which are outside the civil litigation purview. The non-disclosure agreement entered into at the commencement of any mediation may also be advantageous to a constructive outcome. Entering mediation does not preclude subsequent civil litigation but the content of exchanges during mediation are made without prejudice to any subsequent litigation. The experience can be time consuming and emotionally challenging for all who are involved.

NHS Resolution’s recent review of mediation presented descriptive evidence which, in their view, supported the application of mediation. The review concluded that mediation is “proven to be an effective forum for claims resolution” where “¾ of cases mediated are settled on the day of mediation or within 28 days”. NHS Resolution claims that “the introduction of mediation is driving cultural change within NHS” with evidence of “benefits for patients, families and NHS staff”. The review found mediation to be applicable for “all types of claims” and it is proposed that it should be “tailored for greater effect at an earlier stage in the lifecycle of the claim”. The report concludes there is an “underuse of mediation for personal injury claims and costs disputes” and that the benefits of “mediation in these areas should be explored further”. This report offers an optimistic interpretation of evolving descriptive data, which has not been subject to methodological review for independent publication. These claims are far reaching and justify a formal research programme to explore the hypothesis: *mediation as an intervention in the early stage of clinical disputes reduces the number of cases that progress to legal proceedings*.

## Take home messages

- There is no doubt that engagement with the medico-legal aspects of clinical practice is highly instructive, has stimulated major national initiatives to accelerate diagnosis through the HeadSmart campaign, the current Child Cancer Smart campaign, and stimulated research into the mechanisms of brain injury and their mitigation. The risk of cancer for a child is present throughout their early life and so the practitioners seeing children have a duty to keep this risk in their minds.
- The size of compensatory awards in these cases justify careful consideration by health planners to augment systems for their mitigation through practitioner training, public and professional awareness programmes and health services research with linked Quality Improvement programmes targeting raised awareness and practice change.
- It is quite possible that data collected by NHS Resolution and the Medical Defence organisations could, with appropriate anonymity and protection, be usefully analysed and deployed as an adjunct to professional education with the intention of reducing the risk of recurrence.
- Avoidable sudden death of a child due to a treatable cancer is always tragic and needs investigation and honest explanation as a starting point for assisting the family to deal with their grief. Whether there is real evidence to justify adoption of the strategies involving early bereavement support, independent SUIIs, coronial investigations, together with effective mediation as a preferred route of conflict resolution than the civil litigation process, remains to be demonstrated.
- We propose that such approaches could be explored using research methods to support the establishment of new evidence-based guidelines and programmes to monitor the impact of evolving practice. The high personal costs for the affected children with cancers and their families as well as the pecuniary consequences of awards for the NHS and clinical negligence indemnifiers would justify this.

## What is known:

- Childhood cancers present with complications which threaten life and disability and are a common concern about standards of practice for patients and their families.
- Where a breach of duty is identified, legal redress through litigation for resolution of concerns is the route offered by lawyers.
- Expert witness work seeks clinical opinion for breach of duty and clinical consequences linked to the breach.

## What this study adds:

- Delays in diagnosis of brain or spinal tumour are the commonest reason for legal challenge where paediatricians are the main focus for raising concern and has been the focus of successful health campaigns (www.headsmart.org.uk) to change practice.
- The legal process can award substantial damages where a breach of duty is identified and avoidable serious brain or neurological injury results in serious consequences for children with long lives.

## Supplemental Material

sj-pdf-1-mlj-10.1177_00258172221099077 - Supplemental material for The costs of avoidable injury from childhood cancer: Litigate or mediate? Click here for additional data file. Supplemental material, sj-pdf-1-mlj-10.1177_00258172221099077 for The costs of avoidable injury from childhood cancer: Litigate or mediate? by David A Walker and Jonathan Punt in Medico-Legal Journal
