# Supplementary material for: The costs of avoidable injury from childhood cancer: Litigate or mediate?
Source: Med Leg J. 2022 Sep 22;90(4):182–8. doi: 10.1177/00258172221099077 (PMC9716385; doi:10.1177/00258172221099077)
Supplement: sj-pdf-1-mlj-10.1177_00258172221099077 - Supplemental material for The costs of avoidable injury from childhood cancer: Litigate or mediate? [file sj-pdf-1-mlj-10.1177_00258172221099077.pdf]

Appendix: Supplementary material

AUTHORS' EXPERIENCE:
JP was a paediatric neurosurgeon for his first career. A second career emerged from experience of medicolegal work as an expert witness, which took him through legal training to the Bar (Called 2005) where he established a practice in Clinical Negligence and Coronial Law until retiring in 2019. DAW is a clinical academic in paediatric oncology. He has written medical reports concerned with breach of duty and causation related to child cancer practice and brain tumours in particular. Together we have contributed to medical research and clinical development in the fields of neurosurgery and paediatric neuro-oncology.

Table 1: Topics, practitioners, possible breach and causation and proceedings in a review of 35 consecutive expert review reports over 5 years

**Brain tumour diagnostic delay**		**14**	**Practitioners**		**41**
Cerebellum	7		Paediatricians	18	
Hypothalamus	6		MDT Hospital Network	9	
Cortical	1		Radiologist	6	
**Spinal cord compression**		**6**	Paediatric surgeon	3	
Ewing's tumour	1		Ophthalmologist / optician	1	
Neuroblastoma	2		Health visitor	1	
Sacrococcygeal teratoma	1		Neurosurgeon	1	
Vascular tumour	1		Orthopaedic surgeon	1	
High grade glioma	1		Child psychiatrist	1	
**Diagnostic delay other tumour types**		**7**			
Langerhans Cell Histiocytosis (LCH)	1		**Possible Breaches**		
			No Breach identified	4	
Hodgkin's disease	2		Delayed brain scanning	12	
Thyroid cancer	1		Poor / inadequate recording / examination	4	
Hepatoblastoma	1		Misinterpretation of imaging	4	
Synovial cell sarcoma	1		Delay in starting investigation	3	
Adrenocortical carcinoma	1		MDT judgement	2	
**Specialist management.**		**5**	Clinician judgement	2	
Surgical management of hypothalamic astrocytoma	2				
Delayed diagnostic process	1		**Possible causation**		
Imaging error	1		No causation	6	
MDT judgement error	1		Avoidable death	3	
**Treatment complications**		**2**	Shortened life expectancy	5	
Cerebellar mutism syndrome	1		Reduced vision / blind	5	
Treatment related global brain damage and death	1		Hydrocephalus brain damage	5	
			Paraplegia	4	
			Double incontinence	5	
			Serious drug toxicity	5	
			Delay in Rx	1	

**Proceedings**	
Commissioned expert report for Hospital Trust	1
Joint expert meeting between claimant and defendant	2
Court proceedings for award of damages	1
Coroner's court	
Conference with experts and counsel	12
**Outcomes**	

Cases concluded	8
Ongoing / undisclosed	27

Calculation of Damages
Readers from non-legal disciplines should understand a number of factors. When considering the overall financial cost of clinical negligence cases, it is important to distinguish between Costs and Damages. The former include, *inter alia*, the fees of legal representatives and independent experts, charges from the Courts in respect of the issue of proceedings, and charges that might be imposed by NHS Trusts and GPs for provision of documents. The purpose of Damages, as in all species of Personal Injury litigation, is to provide the remedy required to place the injured party in as close a position as possible had the injury not occurred. In reality, this will almost always be provided by pecuniary compensation. Damages fall into two broad categories: General Damages and Special Damages. Any discussions of the rising levels of Damages in clinical negligence settlements should take into consideration the effect of the Discount Rate and note that it is set by the governing political executive. It remains to be pointed out that many very high value cases, especially where life expectancy is uncertain, are not settled on a lump sum basis, but with a combination of a lump sum to cover Past Losses and immediate needs, for example for accommodation and some equipment, plus annual Periodic Payments for Life.

Costs
As the onus lies on the Claimant to prove her/his case, the Claimant will necessarily incur Costs from the outset of investigation of the case. There was a relatively short period during which a successful Claimant in a Clinical Negligence case could recover Success Fees and the cost of After-The-Event (“ATE””) insurance premiums from the unsuccessful Defendant. This undoubtedly added to the financial burden on the NHS and on the indemnifiers of GPs and private practitioners. These additional costs arose in cases undertaken by Claimants’ solicitors and barristers under Conditional Fee Agreements (“CFAs”). It is important to remember that such circumstances were a product of legislation by Parliament, as provided for by the Access to Justice Act 1999 which removed legal aid from personal injury cases, meaning that CFAs became the inevitable route to seeking justice for the Claimant(1). Under the Legal Aid, Sentencing and Punishment of Offenders Act 2012 (“LASPO 2012”), neither ATE insurance premiums nor Success Fees have been recoverable from an unsuccessful Defendant in Clinical Negligence cases under CFAs entered into on or after 1 April 2013.

General Damages are an award of money in respect of injury to the body and/or mind of the injured party, and, in fatal cases, also include the statutory bereavement award that is provided to a strict class of persons under the Fatal Accidents Act 1976, as amended. Save for the latter, which is determined by the Lord Chancellor from time to time, (currently £15,120 for deaths arising from personal injury occurring on or after 1 May 2020(2)), there is no level of General Damages that is fixed in Law. Such sums recoverable by way of General Damages are therefore necessarily imprecise and vary from case to case in a manner that may appear to be arbitrary. Some approximate indication of the level of General Damages that might be recovered can be obtained from the range of sums set out in the 15th edition of “*The Judicial College Guidelines”*, as used in this article for illustration. Even at the highest level, General Damages may seem parsimonious in proportion to the injury suffered despite the 10% uplift on account of Judgment having been given after 1 April 2013, as set out *below(3)*:

**Injury**	**Range of Award**
Tetraplegia (quadriplegia)	£304,630 to £379,100
Paraplegia	£205,580 to £266,740
Very severe brain damage	£264,650 to £379,100
Moderate brain damage	£140,870 to £205,580

Special Damages must be itemised, assessed with precision, and supported by expert opinion and pertinent documentation. The value of the claim provided for by the experts instructed on behalf of the Claimant, as set out in the Claimant's Schedule of Loss and Damage, is usually higher than that in the Defendant's Counter-Schedule. It must also be understood that the vast majority of successful cases are settled by negotiation between the parties, often at Joint Settlement Meetings with legal representation on both sides. Such settlements will take the litigation risks into account as assessed by the parties' legal advisers: an effect of this is that settlements may be reached at levels substantially lower than the sum contended for by the Claimant, and a range of settlements falling between 40% to 70% of the fully pleaded value of the Claimant's Schedule is well-recognised. Many Out of Court settlements are compromised on account of the litigation risks and without any, or any full, admissions of breach of duty or causation on the part of the Defendant. A further factor complicating the paucity of cases in which Damages are determined by a Judge at Trial, is the selective nature of the minority of those cases that are reported in the legal literature. Furthermore, there are very few cases that are directly equivalent as to their facts, with the effect that the reported cases can only provide very approximate comparators.

The major heads of loss under Special Damages will frequently be the costs of past and future care, accommodation, equipment, professional Case Management, and, in cases where the Claimant lacks mental capacity, the costs arising from the Court of Protection. If there are ongoing therapeutic needs then the cost of same in the private sector can be sought as the Claimant is not required to rely upon services provided by the State. This has particular relevance in cases of children suffering brain or spinal injury. NHS rehabilitation provision for paediatric neuro-oncology patients remains inadequate through limitations of resource, although the expertise in its planning and implementation are well supported by expert professionals(4): follow-up and ongoing healthcare for the growing numbers of adult survivors of childhood cancers of all types is also an incompletely met need limited by resources(5). It is important to have in mind the fact that only those injuries, and the needs flowing therefrom, that were caused or materially contributed to by the alleged negligence will be compensated for in Damages. There are many cases in which some degree of injury or domain of impairment will have occurred even with appropriate management. In many cases there will be complex issues of Causation. Whereas expert opinion may assist the Court in the field of disputed medical causation, issues of factual causation and legal causation will lie in the province of the Judge and legal argument.

Life Expectancy

It is important to be aware that the intention of any Future Damages is that they will be sufficient to provide for the Claimant's needs arising from, and attributable to, the avoidable injury for as long as such needs will persist: frequently these will be lifelong and it follows that, whereas the Claimant should not be over-compensated, such Damages recovered must be protected against inflation. Inevitably, life expectancy will have a major impact on quantum of Damages, especially when the Personal Injury Discount Rate is taken into account. The Discount Rate is determined by the Lord Chancellor from time to time for the purposes of taking into account the fact that the Claimant is in early receipt of her/his future expenditure, and that the reasonably prudent Claimant will be in a position to make secure investments with any lump sum payments received in compensation. For many years the Discount Rate lay at 2.5%, a level that persisted long after it was an accurate reflection of the yield to be expected from safe investments: the effect was to benefit Defendants, including the NHS, the reason being that Damages in respect of Future Losses and Expenses were kept down. In February 2017 the then Lord Chancellor belatedly amended the Discount Rate in England and Wales to minus 0.75%(6), effective from 20 March 2017 and was further amended to minus 0.25% on 15 July 2019, effective from 5 August 2019(7). The inevitable effect was a very substantial increase in the cost of claims, the reason being that a Discount Rate set at a minus

number has a dramatic effect on Damages for Future Losses. By way of example, the case of a male who has suffered an avoidable brain or spinal injury and for whom the cost of his future needs is £250,000 *per* annum is considered. If said person is aged 15 years at the date of Settlement, and he has a full life expectancy as *per* the projection in the Office of National Statistics Life Expectancy Tables, the considerable effect of various Discount Rates, as derived from Ogden Table 1 (8), on a Lump Sum Settlement can be seen set out Table 2:

Table 2

Date of Settlement	Cost of Future Care (£pa)	Discount Rate	Multiplier for Life	Value of Claim (£)
Up to 20/3/17	250,000	2.5%	33.14	8,285,000
20/03/17 - 5/8/20	250,000	- 0.75%	96.52	24,130,000
From 5/8/20	250,000	0.25%	79.02	19,755,000

In many cases of negligently managed childhood brain tumour with serious acquired brain injury, there will be issues that will lead to a reduced life expectancy, either flowing from the avoidable injury or arising from the inherent nature of the tumour: but even then, taking the example of annual care valued at £250,000, the substantial impact on the resultant applicable multipliers, as derived from Ogden Table 36 (8), and thus on the Lump Sum cost, of the three various Discount Rates that have been in effect from before March 2017 to, and from, August 2020, is illustrated in Table 3:

Table 3

Life expectancy	Discount Rate 2.5%		Discount Rate - 0.75%		Discount Rate - 0.25%	
	Multiplier	Cost (£)	Multiplier	Cost (£)	Multiplier	Cost (£)
10 years	8.86	2,215,000	10.39	2,597,500	10.13	2,532,500
20 years	15.78	3,945,000	21.58	5,395,000	20.51	5,127,500
40 years	25.42	6,355,000	46.68	11,670,000	42.07	10,517,500
60 years	31.29	7,822,500	75.84	18,960,000	64.74	16,185,000

Whether compensatory payments fall to the NHS, or to the malpractice indemnifiers of GPs, other private medical practitioners, or private health care facilities, is a matter of Law; the successful Claimant is not required to rely upon services provided by the State. Whereas it is open to the Claimant to deploy her/his Damages to seek paid services from any provider, it is unsurprising if an injured party chooses not to place reliance upon a State provider that has failed her/him already, even if a mechanism existed by which a successful Claimant could purchase services from the State and chose to do so. Indeed, it would be frankly unconscionable if a Claimant who was in funds could purchase a service at the expense of the ordinary NHS patient, especially in an underfunded and over-stretched service that struggles to provide for those who labour under non-avoidable impairments. Further, many of the more costly items required cannot be provided by the Defendant: suitable accommodation is a notable example. The expertise for rehabilitation within the NHS sector, particularly in paediatrics is integrated with education and social care. There is no mechanism for using compensatory funding within State funding streams to recycle the money within the State system, except by relieving the state of the costs of equipment purchase where the family chooses. This may place the family as major decision makers in fields where they lack expertise, although they may choose to appoint a personal injury manager with the Damages awarded. The State's process of allocating resource for providing suitably coordinated care for an

injured child, once she/he enters adult life is often a protracted and complex negotiation with uncertain outcomes in planning for their needs, over the decades ahead.

EXAMPLES OF COMPENSATORY CALCULATIONS

In any case of alleged clinical negligence, it will be important to distinguish between injuries that have been caused or materially contributed to by the alleged breach of duty, as opposed to any injuries that would have probably arisen absent said failure. For example, an implanted hydrocephalus shunt may have been required in any event in a younger child with a benign cerebellar astrocytoma: a degree of hypothalamic dysfunction and some visual impairment may have been inevitable in a case of craniopharyngioma undergoing the more recent shift towards more conservative surgery despite reduction in hormone deficiencies(5) and the avoidance of very severe visual loss that would have been avoided with earlier, non-negligent, diagnosis. A degree of neuropsychological impairment on account of chronic hydrocephalus may not have been avoidable with proper management, whereas the injury attributable to the negligent development of an acute hydrocephalic attack will be grounds for compensation. Necessary radiation therapy to the neuraxis in the developing phase of life will always carry risks of learning difficulties, neuroendocrine deficiencies, and secondary cancers. Necessary laminectomy for an extensive intramedullary spinal cord tumour may result in later spinal deformity. There may also be cases where there are important interactions between impairments that are inherent to the disease and/or its proper management and the effects of negligence. For example, the child who would have been capable of independent life if she/he was only mildly ataxic following an adequately managed benign cerebellar tumour will be disproportionately disadvantaged by avoidable visual loss and learning difficulties arising from avoidable delay in recognition and relief of hydrocephalus. By contrast, the child who has reduced neuropsychological function resulting from necessary whole brain radiation therapy to treat a medulloblastoma, will be at increased disadvantage from avoidable visual loss on account of difficulties with rehabilitation as a visually-impaired person.

Acute on chronic hydrocephalus on a background of a benign mass lesion

The level of Damages will be determined by the extent and severity of the avoidable component of the acute event. Whereas it may be argued that the chronic component could inevitably have been associated with some degree of neuropsychological impairment, especially in the domain of memory, it may well be able to establish that visual loss, motor impairment, and major intellectual loss, at levels that care will be required for life, flow from the alleged negligence.

ABDUL HOSN v TRUSTEES OF THE ITALIAN HOSPITAL (1987)

A 19 year old man with considerable academic potential suffered severe permanent brain damage on account of negligent post-operative management of hydrocephalus in a private hospital following excision of a colloid cyst. The Claimant had severely reduced neuropsychological function and he would require lifetime day and night-time care, in addition to accommodation needs and physiotherapy, *inter alia*. Later in life the Claimant would require residential care. At the date of Trial the Claimant was aged 23 years and his life expectancy was 36 to 41 years. The damages were awarded by a Judge at Trial, and the case was the first clinical negligence case in England and Wales in which damages exceeded £1 million. When adjusted for Retail Price Index ("RPI"), the damages awarded would equate in 2020 to a total of £2,631,313, of which General Damages would have comprised £248,582 (*Abdul Hosn v Trustees of The Italian Hospital (1987).* Kemp/Lawtel, Document Number AM0076336). The cost of Future Care required would certainly have resulted in much higher damages if the case had been settled in 2020 when the current Discount Rate of minus 0.25% would have applied.

Hypothalamic injury in a benign tumour

X (BY HIS FATHER AND LITIGATION FRIEND) v (1) CUMBRIA AND LANCASHIRE STRATEGIC HEALTH AUTHORITY (2) LANCASHIRE TEACHING HOSPITALS NHS FOUNDATION TRUST (2009)
When aged seven years a child who had been under observation in a hospital refraction clinic experienced a dramatic reduction in visual acuity which was not investigated. Two years later the child suffered severe headaches, vomiting, impaired balance and visual loss., at which point he was found to have a pilocytic astrocytoma involving the anterior visual pathways. The child underwent surgical reduction in tumour bulk, radiation therapy and insertion of a ventricular shunt: the latter was followed by extensive wound complications. The child suffered severe visual loss, executive dysfunction, cognitive impairment, memory difficulties and endocrine deficiencies requiring lifelong replacement hormone therapy. It is noteworthy that it was contended that radiation therapy would not have been avoided with competent management and earlier diagnosis, and the Defendants made no admissions regarding the acquired brain injury. An Out of Court settlement was reached when the Claimant was aged 22 years and was approved by a Judge. Total damages, adjusted for RPI, would now equate to £2,654,843, including General Damages equating to £314,389. Again, the cost of Future Care required would almost certainly have resulted in much higher damages with settlement in 2020 when the current Discount Rate of minus 0.25% would have applied (*X (by his Father and Litigation Friend) v (1) Cumbria and Lancashire Strategic Health Authority (2) Lancashire Teaching Hospitals NHS Foundation Trust (2009).* Lawtel, Document Number AM0201498).

In an unreported case a child with a low grade pilocytic astrocytoma in the region of the optic chiasm and hypothalamus underwent an operation at the age of three years on account of an increase in the size of the tumour on serial MRI. Vision was stable and there was no RICP. There were cutaneous and neuroradiological features of neurofibromatosis type 1. Radical resection of the tumour was undertaken with the effects that the child suffered brain injury manifesting as a hypothalamic syndrome with severe permanent behavioural disturbance, neuroendocrine dysfunction and cognitive impairment. Expert opinions were that less extensive resection would have been the correct approach. The Defendant NHS Trust admitted liability to compensate the child for the consequences of the radical resection, including the hypothalamic damage. Lifetime care included 24-hour resident care from psychiatric-trained staff and accommodation, with a long life expectancy. When the Claimant was aged 23 years, an Out of Court Settlement was achieved with Damages taking the form of a lump sum plus Periodical Payments for life: the settlement equated to a sum exceeding £26 million.

Spinal cord compression due to a curable tumour causing paralysis and double incontinence
An unreported case of acquired tetraparesis with incontinence occurring at age 15 years in a child with life expectancy to her eighth decade was valued at £290,000 with respect to General Damages and £8.9 million in Special Damages. The major heads of Special Damages were the costs of future care and assistance, accommodation and equipment. The claim was settled at a Joint Settlement Meeting at which a lump sum of £1.9 million was agreed with periodical payments commencing at £100,000 *per annum*, rising to £125,000 *per annum* after 15 years, and then after a further 10 years to £165,000 *per annum* for life.

1. Litigation Futures. A Guide To The Jackson Reforms And Civil Litigation Costs 2018 [cited 2020 18 November 2020]. Available from: https://www.litigationfutures.com/features/jackson-reforms-a-complete-guide.
2. Damages for Bereavement (Variation of Sum) (England and Wales) Order Ministry of Justice; 2020.
3. Judicial College Guidelines for the Assessment of General Damages in Personal Injury Cases 15th Edition. 15th ed. Oxford: Oxford University Press; 2019.

4. Treadgold B, Kennedy C, Spoudeas H, Sugden E, Walker D, Bull K. Paediatric neuro-oncology rehabilitation in the UK: carer and provider perspectives. BMJ Paediatr Open. 2019;3(1):e000567. PubMed PMID: 31909222. Pubmed Central PMCID: PMC6937011. Epub 2020/01/08.
5. Gan HW, Spoudeas HA. Long-term follow-up of survivors of childhood cancer (SIGN Clinical Guideline 132). Arch Dis Child Educ Pract Ed. 2014 Aug;99(4):138-43. PubMed PMID: 24639409. Pubmed Central PMCID: PMC4112452. Epub 2014/03/19.
6. New discount rate for personal injury claims announced. Ministry of Justice; 2017.
7. Lord Chancellor announces new discount rate for personal injury claims. Ministry of Justice; 2019.
8. Actuarial Tables With Explanatory Notes for use in Personal Injury and Fatal Accident Cases: 8th Edition. In: Department As, editor. 8th ed: Crown Copyright; 2020.
